# Podocytopathies associated with familial partial lipodystrophy due to *LMNA* variants: report of two cases

**DOI:** 10.20945/2359-4292-2023-0204

**Published:** 2024-05-10

**Authors:** Maria Julia Morguetti, Precil Diego Miranda de Menezes Neves, Ilana Korkes, Wallace Stwart Carvalho Padilha, Lectícia Barbosa Jorge, Andreia Watanabe, Elieser Hitoshi Watanabe, Denise Maria Avancini Costa Malheiros, Irene de Lourdes Noronha, Sergio Atala Dib, Luiz Fernando Onuchic, Regina S. Moisés

**Affiliations:** 1 Universidade Federal de São Paulo Escola Paulista de Medicina Divisão de Endocrinologia São Paulo SP Brasil Divisão de Endocrinologia, Escola Paulista de Medicina, Universidade Federal de São Paulo, São Paulo, SP, Brasil; 2 Universidade de São Paulo Faculdade de Medicina Divisões de Nefrologia e Medicina Molecular São Paulo SP Brasil Divisões de Nefrologia e Medicina Molecular, Faculdade de Medicina, Universidade de São Paulo, São Paulo, SP, Brasil; 3 Universidade Federal de São Paulo Escola Paulista de Medicina Divisão de Nefrologia São Paulo SP Brasil Divisão de Nefrologia, Escola Paulista de Medicina, Universidade Federal de São Paulo, São Paulo, SP, Brasil; 4 Universidade de São Paulo Faculdade de Medicina Divisão de Nefrologia São Paulo SP Brasil Divisão de Nefrologia, Faculdade de Medicina, Universidade de São Paulo, São Paulo, SP, Brasil; 5 Universidade de São Paulo Faculdade de Medicina Divisões de Nefrologia Pediátrica e Medicina Molecular São Paulo SP Brasil Divisões de Nefrologia Pediátrica e Medicina Molecular, Faculdade de Medicina, Universidade de São Paulo, São Paulo, SP, Brasil; 6 Universidade de São Paulo Faculdade de Medicina Departamento de Patologia São Paulo SP Brasil Departamento de Patologia, Faculdade de Medicina, Universidade de São Paulo, São Paulo, SP, Brasil; 7 Universidade de São Paulo Faculdade de Medicina Departamento de Clínica Médica São Paulo SP Brasil Departamento de Clínica Médica, Faculdade de Medicina, Universidade de São Paulo, São Paulo, SP, Brasil

## Abstract

Lipodystrophies are characterized by complete or selective loss of adipose tissue and can be acquired or inherited. Familial partial lipodystrophy (FPLD) is a hereditary lipodystrophy commonly caused by mutations in the *LMNA* gene. Herein, we report two cases of FPLD associated with podocytopathies. Patient 1 was diagnosed with FPLD associated with the heterozygous p.Arg482Trp variant in *LMNA* and had normal glucose tolerance and hyperinsulinemia. During follow-up, she developed nephrotic-range proteinuria. Renal biopsy was consistent with minimal change disease. Patient 2 was diagnosed with FPLD associated with a *de novo* heterozygous p.Arg349Trp variant in *LMNA*. Microalbuminuria progressed to macroalbuminuria within 6 years and to nephrotic range proteinuria in the last year. He remained without diabetes and with hyperinsulinemia. Renal biopsy revealed focal segmental glomerulosclerosis not otherwise specified. This report provides further evidence of variable features of lipodystrophy associated with *LMNA* variants and the importance of long-term follow-up with evaluation of kidney dysfunction.

## INTRODUCTION

Lipodystrophies are acquired or inherited disorders characterized by localized or generalized loss of subcutaneous adipose tissue (1). They are associated with metabolic complications – such as insulin resistance, diabetes mellitus, dyslipidemia, and hepatic steatosis – whose severity depends on fat loss. Renal diseases have also been described in generalized (acquired or inherited) and acquired partial lipodystrophies (2-4). Previous reports of patients with generalized lipodystrophies have shown associations with proteinuria and different spectrum of morphologic findings at kidney biopsy, including membranoproliferative glomerulonephritis (MPGN), focal segmental glomerulosclerosis (FSGS), and less frequently, diabetic nephropathy (2,3). In an international chart review study, nephropathy was identified in 59.7% of patients with generalized lipodystrophy (5), while in patients with acquired partial lipodystrophy, the estimated prevalence of histologically documented MPGN was 22% (6). However, renal disease is rare in familial partial lipodystrophy (FPLD), with few cases reported so far (7-12). Among FPLDs, the Dunnigan variety or type 2 (FPLD2) is the most prevalent subtype (1). It is an autosomal dominant condition characterized by gradual loss of fat from limbs and trunk with face and neck fat accumulation and onset around puberty. Notably, FPLD is caused by pathogenic mutations in *LMNA*, the gene encoding lamins A and C, components of the nuclear lamina (13). Among the *LMNA* variants associated with lipodystrophy, a distinct phenotype has been observed in carriers of the p.R349W. In the few such cases, a different pattern of fat distribution and association with kidney and cardiac complications have been observed (10,11,14-17).

Podocytopathies are a group of glomerular diseases manifested under the histological forms of minimal change disease (MCD), FSGS, or collapsing glomerulopathy (18,19). The pathogenesis of podocytopathies is currently divided into four forms: a) mediated by permeability factors; b) mediated by cytokine direct toxicity, associated or not with apolipoprotein L1 (*APOL1*) high-risk genotypes; c) mediated by hyperfiltration; and d) caused by genetic disorders (18). The genetic causes are associated with mutations in podocyte genes that encode proteins that participate in anchoring pedicels between themselves (slit membrane) and the basal membrane, mitochondrial proteins involved in coenzyme Q10 biogenesis, proteins associated with binding and regulation of the actin cytoskeleton, proteins that form the basal membrane (18,19), and proteins that are components of the nuclear envelope, such as lamin A and lamin C, products of the *LMNA* gene (10,20).

Here, we present the clinical course of two patients with FPLD harboring *LMNA* variants who developed podocytopathies.

## CASES PRESENTATION

### Patient 1

The proband is a 36-year-old woman referred for hypertriglyceridemia and gradual fat loss from limbs since the age of 10 years. At the age of 18 years, fat accumulation occurred in the neck, submental, and supraclavicular regions. Her mother and two sisters displayed a similar phenotype. Her mother had diabetes mellitus, dyslipidemia, and premature coronary disease (coronary artery bypass at the age of 53 years). One sister was diagnosed with polycystic ovary syndrome, presenting menstrual disorder and hirsutism. The other sister had no metabolic manifestations.

Physical examination showed a body mass index of 25.4 kg/m^2^ (weight of 69.2 kg and height of 165 cm), loss of subcutaneous adipose tissue in the arms, legs, and gluteal region but sparing the face, and fat accumulation in the submental region. Acanthosis nigricans in axillary and nuchal regions, and appearance of increased muscularity in the calves and hypomastia were also present. Biochemical investigation showed hypertriglyceridemia, normal glucose tolerance with hyperinsulinemia, and normal renal and hepatic functions (Table 1). Abdominal ultrasound revealed hepatic steatosis. Direct Sanger sequencing of the *LMNA* gene revealed the heterozygous variant p.Arg482Trp (NM_170707.4:c.1444C>T), confirming the diagnosis of FPLD2. The three family members with clinical features of lipodystrophy displayed the same variant.

**Table 1:** Patients' biochemical parameters

Parameters		Patient 1	Patient 2
Oral glucose tolerance test		
	Fasting plasma glucose (mg/dL)	71	92
	2-hour glucose (mg/dL)	118	109*
Fasting plasma insulin (µU/mL)		70.3	37
Glycated hemoglobin (%)		5.4	5.5
Urea (mg/dL)		27	30
Creatinine (mg/dL)		0.63	0.54
Total cholesterol (mg/dL)		201	158
LDL cholesterol (mg/dL)		82	94
HDL cholesterol (mg/dL)		32	34
Triglycerides (mg/dL)		701	204
AST (U/L)		31	-
ALT (U/L)		38	-

Abbreviations: ALT, alanine aminotransferase; AST, aspartate aminotransferase; HDL cholesterol, high-density lipoprotein cholesterol; LDL cholesterol, low-density lipoprotein cholesterol. * 2-hour postprandial glucose

**Figure 1:**
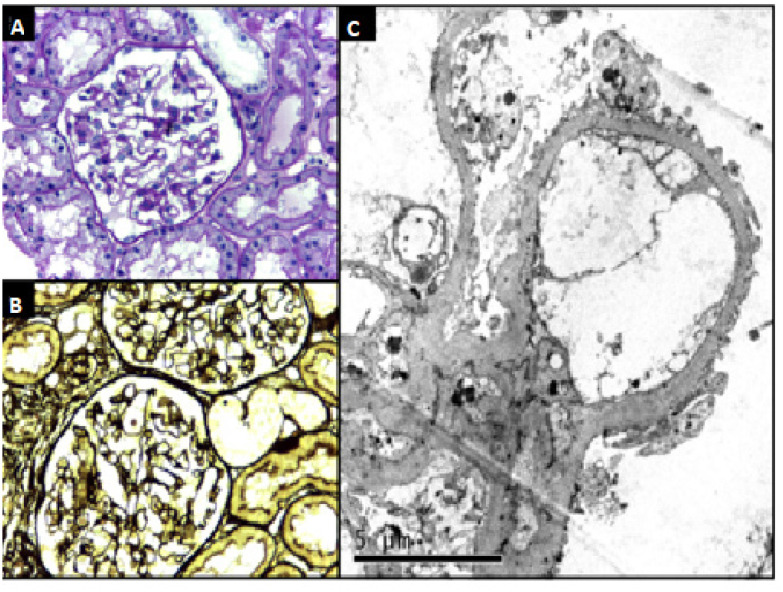
Patient 1. (**A** and **B**) Renal biopsy showing glomeruli with normal aspects on light microscopy (A, HE, x400; B, PAMS, x400) (**C**) but with diffuse collapse of foot processes consistent with genetic podocytopathy on electron microscopy.

At the age of 33 years, the patient developed nephrotic-range proteinuria (4.18 g in 24-hour urine collection) without signs of nephrotic syndrome. Complementary laboratory investigation showed serum creatinine of 0.52 mg/dL (estimated glomerular filtration rate [eGFR] calculated with the CKD-EPI equation of 126 mL/min/1.73 m^2^), serum albumin of 3.9 g/dL, negative serologies for hepatitis B, hepatitis C, and HIV, negative findings for antinuclear antibody, anti-double-stranded DNA, antineutrophil cytoplasmic autoantibodies, rheumatoid factor, and normal serum C3 and C4 complement fractions. A kidney biopsy was performed, and light microscopy showed eight glomeruli, of which one was globally sclerotic, and the others had normal cellularity and capillary loops with regular contours. Small and sparse tubular atrophic foci and interstitial fibrosis were observed, without vascular abnormalities. No significant deposits were detected by immunofluorescence. Electron microscopy revealed degenerative podocyte alterations with diffuse foot process effacement (>80%) but absence of electrodense deposits, a pattern consistent with MCD (Figure 1). The patient is currently on an angiotensin II–receptor blocker, with 1.18 g/day of proteinuria and eGFR (CKD-EPI equation) of 85 mL/min/1.73 m^2^. Of note, the patient's mother and two sisters did not present abnormal proteinuria. To exclude potential variants in other known genes associated with proteinuric nephropathies, the patient was submitted to whole exome sequencing (WES). The finding of no such variants ruled out other potential genetic causes of MCD.

### Patient 2

**Figure 2:**
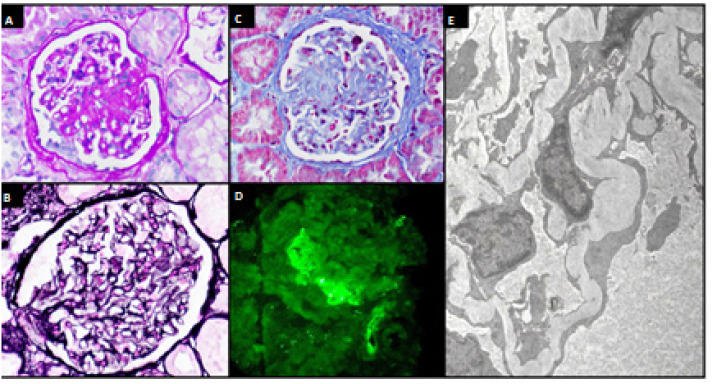
Patient 2. (**A**, **B**, and **C**) Renal biopsy showing glomeruli with areas of focal segmental sclerosis (A, PAS, x400; B, Masson's trichrome, x400) without changes in the basement membrane (C, PAMS, x400). (**D**) Immunofluorescence showing IgM +1/+3 and C3 +2/+3 positivity with mesangial and focal distribution. (**E**) Electron microscopy showing diffuse collapse of foot processes consistent with genetic podocytopathy (x3700).

This 33-year-old man had his clinical case previously reported (15). Briefly, subcutaneous fat loss in upper and lower limbs was observed, including hand palms and foot soles, allowing observation of visible veins. Micrognathia and short stature were also present. Direct Sanger sequencing revealed a *de novo* heterozygous p.Arg349Trp (NM_170707.4:c.1045C>T) variant in *LMNA*. He remained without diabetes (glycated hemoglobin of 5.5%, fasting plasma glucose level 92 mg/dL) with hyperinsulinemia during follow-up. Laboratory data are shown in Table 1. Microalbuminuria was first noted at the age of 19 years (33 mg/g creatinine) and progressed to overt albuminuria (465 mg/g creatinine) over the following 6 years. An angiotensin-converting-enzyme inhibitor was initiated but had to be suspended 3 years later due to hypotension. Non-nephrotic proteinuria remained stable over the years and progressed to the nephrotic range during the last year (4.2 g/day). The patient remained normotensive, without edema, and with serum creatinine of 0.73 mg/dL (eGFR of 123 mL/min/1.73 m^2^); serum albumin of 4.6 g/dL; negative serologies for hepatitis B, hepatitis C, and HIV; negative findings for antinuclear antibody, anti-double-stranded DNA, antineutrophil cytoplasmic autoantibodies, and rheumatoid factor; and normal C3 and C4 complement fractions. A kidney biopsy previously not reported revealed 13 glomeruli, of which three were globally sclerotic and two had segmental mesangial expansion and synechiae (Figure 2A-C), while the remaining glomeruli had normal aspect. Bowman's capsules focally thickened, mild interstitial fibrosis, and tubular atrophy without vascular abnormalities were observed. Immunofluorescence showed IgM and C3 mesangial deposits with segmental and focal distribution (Figure 2D). Electron microscopy (Figure 2E) evidenced trilaminated glomerular basal membrane in some regions, with wrinkling and irregularly thickened areas. Podocytes with focal degenerative changes and extensive foot process fusion were detected in more than 50% of the tufts. These findings were consistent with FSGS not otherwise specified. The patient was started on losartan 50 mg twice a day, with a reduction in the urine protein-creatinine ratio to 2.3 g/g. The patient also developed hearing impairment at the age of 27 years and was diagnosed with mixed aortic valve disease at the age of 29 years, which required valve replacement.

## DISCUSSION

We described two cases of partial lipodystrophy associated with *LMNA* variants in patients without diabetes who developed nephrotic-range proteinuria. Notably, FPLD2 is a heterogeneous disorder associated with metabolic alterations and, in a few reported cases, with glomerular diseases. A case of progressive renal disease in a patient with diabetes and FPLD2 due to *LMNA* p.Arg482Gln has been reported previously; however, as kidney biopsy was not performed, the renal diagnostic was not established (9). Klupa and cols. identified the same variant in a patient with partial lipodystrophy, diabetes mellitus, chronic renal disease, and pulmonary fibrosis. This patient was suspected of having sarcoidosis, but this diagnosis was not confirmed and renal biopsy was not performed (21). Owen and cols. first reported a case of mesangiocapillary glomerulonephritis type 2 without hypocomplementemia or elevated factor C3 nephritic in a patient with diabetes who was heterozygous for the p.Arg482Trp variant (7). Additional reports of FPLD2 associated with codon 482 variants and renal involvement were in patients with diabetes mellitus (12,22). In contrast to these reports, our first case did not manifest diabetes mellitus. To the best of our knowledge, this is the first report of FPLD2 associated with nephrotic-range proteinuria due to MCD. Previously, Jacob and cols. described a pediatric case of nephrotic syndrome due to MCD with loss of subcutaneous fat in the face and trunk after a short febrile illness, however no genetic assessment was performed (23). Since no previous reports have established or confirmed this association, we performed WES to exclude variants that could potentially justify the observed podocytopathy. The lack of such variants supports a causative association in our patient while suggesting incomplete kidney penetrance of this variant since the other affected family members did not present kidney alterations.

The *LMNA* p.Arg349Trp variant, on the other hand, has been associated with a different pattern of lipodystrophy and distinct features such as short stature, some progeroid features, hearing impairment, and heart valve abnormalities (10,16,17). Although the association of the *LMNA* p.Arg349Trp variant with kidney disease is well described (10,11,16), available information is still scarce. Our second case illustrates various manifestations observed in patients with the R349W *LMNA* variant, including the kidney histological profile. In our patient, its onset was insidious over the years and agrees with early reports showing that proteinuria is a frequent feature of this syndrome. Kidney biopsy showed FSGS, as reported in other patients, but it should be noted that thin basement membrane disease and mild focal glomerular mesangioproliferative nephropathy have also been described (11,14). A proposed mechanism by which *LMNA* mutations may lead to glomerulosclerosis involves transforming growth factor beta 1 (TGF-β1). Through alternative splicing, *LMNA* gene encodes lamins A and C. In addition to maintaining the integrity of the nuclear membrane, these isoforms interact with different transcription factors, including sterol response element binding protein 1 (SREBP1). Notably, TGF-β1 is a profibrotic cytokine whose transcriptional response is regulated by SREBP1 (24). Vadrot and cols. showed that the *LMNA* p.R482W mutation impairs LMNA-SREBP1 interactions and upregulates SREBP1 target gene expression (25). Also, transgenic mice overexpressing TGF-β1 develop glomerulosclerosis and a lipodystrophy-like syndrome (26).

In summary, we described two cases of podocytopathies associated with lipodystrophy harboring *LMNA* variants. The first patient displayed an unusual association of nephrotic-range proteinuria due to MCD with FPLD2 and had no other underlying disease or identifiable additional cause. The second case adds to the few reported cases of lipodystrophy due to p.Arg349Trp variant associated with proteinuric nephropathy. Lipodystrophy associated with *LMNA* variants presents with variable features, and long-term, careful follow-ups are essential to assess potential renal disease, even in the absence of diabetes mellitus.
